# The impact of frailty on quality of life among older adults in nursing homes: the mediating role of psychological resilience

**DOI:** 10.3389/fpubh.2025.1692042

**Published:** 2025-10-20

**Authors:** Jiquan Zhang, Wei Qing, Yang Zhou, Jingru Zhou, Fan Xu

**Affiliations:** ^1^Nursing Department, Deyang People's Hospital, Deyang, China; ^2^Science and Education Department, Deyang People's Hospital, Deyang, China; ^3^Oncology Department, Deyang People's Hospital, Deyang, China

**Keywords:** nursing home, older adults, frailty, psychological resilience, quality of life

## Abstract

**Background:**

As China’s population ages, nursing homes are becoming increasingly important in the care system for older adults. However, older adults in nursing homes often face high health risks and challenges to their quality of life (QOL) due to advanced age, disability, and multiple coexisting illnesses. Frailty, as a multidimensional clinical syndrome associated with age, significantly affects the QOL of older adults.

**Objective:**

To investigate the mediating role of psychological resilience in the relationship between frailty and QOL among older adults in nursing homes.

**Methods:**

A cross-sectional survey method was employed, with 302 older adults residing in nursing homes in China from August to November 2022 selected as the study subjects. A questionnaire survey was conducted using a general information questionnaire, the Tilburg Frailty Indicator (TFI), the Connor-Davidson Resilience Scale (CD-RISC 25), and the Medical Outcomes Study 36-Item Short Form Health Survey (SF-36). Data analysis was performed using SPSS 26.0 and its PROCESS macro program.

**Results:**

Frailty among older adults in nursing homes significantly negatively predicted QOL (β = −0.224, *p* < 0.001), and psychological resilience partially mediated the relationship between frailty and QOL, accounting for 41.55% of the total effect. Frailty indirectly affected QOL by reducing psychological resilience (β = 0.093, 95% CI = [−0.150, −0.050]).

**Conclusion:**

Psychological resilience is a key mediating variable between frailty and QOL. Enhancing the psychological resilience of older adults in nursing homes can help mitigate the negative impact of frailty on QOL, providing a theoretical basis and practical guidance for improving the QOL of older adults in nursing homes.

## 1 Introduction

China’s aging population has become increasingly serious. According to statistics from the 2021 National Development Report on Aging (1), as of 2021, the population aged 60 and above in China reached 267 million, accounting for 18.9% of the total population. It is projected that by around 2035, the population aged 60 and above will exceed 400 million. Against this backdrop, the older adult care model is undergoing a profound transformation. Institutional older adult care, as a key pillar of China’s older adult care service system, is increasingly assuming a strategic role (2). However, older adults generally have characteristics such as advanced age, disability, and multiple coexisting diseases (3), due to the limitations of nursing homes’ management models and living environments, residents often face challenges such as restricted activity space, lack of social interaction, and weak family support (4). These factors collectively contribute to higher health risks and challenges to quality of life (QOL) for this group. The World Health Organization (WHO) defines QOL as an individual’s perception of their position in life within the context of the culture and value systems in which they live, and in relation to their goals, expectations, standards, and concerns. It reflects an individual’s subjective experience of their physical condition, psychological functioning, social capabilities, and overall well-being (5, 6). Under the biopsychosocial medical model, QOL has become a core outcome indicator for measuring individual health and well-being, lifestyle, disease intervention, and the effectiveness of older adult care (7). Therefore, assessing the QOL of older adults in nursing homes and conducting an in-depth analysis of the factors that influence it is of great theoretical and practical significance for the precise optimization of health intervention strategies and the improvement of the physical and mental health of this group.

Among the many factors influencing the QOL of older adults in nursing homes, frailty is widely recognized as a key predictive variable. Frailty is defined as an age-related multidimensional clinical syndrome characterized by a decline in physiological system reserve capacity and multisystem dysfunction, leading to weakened ability to maintain homeostasis and increased susceptibility to stress events (8). Related studies show that frailty significantly increases the risk of adverse health outcomes such as delirium, falls, disability, hospitalization, and death (9–11), and directly harms the QOL of older adults (12). Kanwar et al. found that even after controlling for individual factors, frail older adults still had lower QOL and life satisfaction than non-frail older adults (13). A survey of older adults in Sri Lanka also showed that frailty is negatively correlated with QOL: the greater the frailty, the poorer their QOL (14). A systematic review and meta-analysis showed that the association between frailty and lower QOL was clear and often significant across multiple constructs (15). In summary, existing literature consistently indicates that frailty is a key predictor of QOL. Based on this, we propose Hypothesis 1: There is a significant negative correlation between frailty and QOL.

Psychological resilience refers to an individual’s ability to adapt well when faced with adversity, grief, threats, trauma, or other negative experiences. It is a positive psychological trait (16). A large body of research has shown that psychological resilience has multiple positive effects on older adults, including improved ability to perform daily activities, increased happiness and life satisfaction, reduced risk of death, and improved QOL (17–20). A study of older adults hospitalized for chronic diseases showed that psychological resilience has a significant impact on the QOL of older adults (21). A survey conducted by Zheng et al. on 418 community older adults showed that psychological resilience is positively correlated with QOL (22). Meanwhile, research shows that frailty is negatively correlated with psychological resilience (23). A three-year longitudinal cohort study showed that higher levels of psychological resilience were associated with a reduction in frailty, particularly among those with initially higher frailty levels. Furthermore, changes in psychological resilience were associated with the progression of frailty, demonstrating a nonlinear association (24). In addition, the stress process theory points out that when individuals encounter stressful life events, they will actively respond to stress by mobilizing internal and external protective resources of the body, thereby alleviating the adverse effects of stressful life events on physical and mental health. The core mechanism lies in that high psychological resilience reduces stress perception and promotes adaptive coping strategies, thereby improving health outcomes (25). This resonates with the Resilience in Illness Model (RIM), which emphasizes that psychological resilience is a key buffer variable between illness stress and health outcomes (26). Therefore, based on relevant theories and empirical research, this study considers frailty as a stressful event faced by older adults, psychological resilience as a core protective resource, and QOL as an outcome variable, and proposes Hypothesis 2: Psychological resilience may be a mediating variable between frailty and QOL.

**Figure 1:**
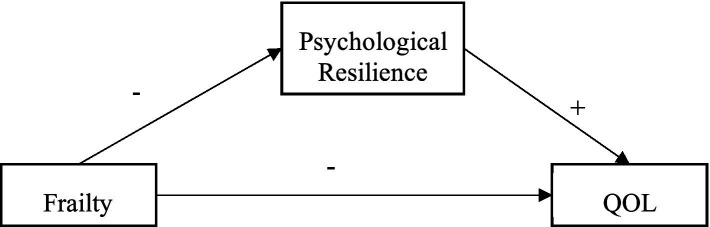
The proposed mediated model. QOL, quality of life.

Currently, studies have explored the relationship between frailty, psychological resilience, and QOL in bladder cancer participants (27) and community residents (28) but no studies have explored the possible mediating role of psychological resilience between frailty and QOL. This study is based on the stress process theory and proposes research hypotheses to explore the mediating role of frailty and QOL among older adults in nursing homes (Figure 1). The purposes of this study were to (a) examine whether frailty can significantly predict QOL; (b) examine whether psychological resilience mediates the relationship between frailty and QOL.

## 2 Methods

### 2.1 Participants

This study was designed as a cross-sectional survey; convenience sampling was used to select older adults residing in nursing homes in Deyang City, Sichuan Province, China, from August to November 2022 as the study subjects. Inclusion criteria were: (1) age ≥ 60 years; (2) nursing home residence time ≥ 12 months; (3) informed consent and voluntary participation in the survey. Exclusion criteria were: (1) cognitive impairment and consciousness impairment (Such as Alzheimer’s disease, schizophrenia, and bipolar disorder); (2) language communication barriers, and other conditions that prevented cooperation. Based on the linear regression model, the required sample size was calculated using G*Power software (Version 3.1) (29), with a power of 0.95 and an alpha of 0.05. According to Cohen’s standard (30), the effect size f^2^ was assumed to be 0.15 (moderate effect size), and the number of independent variables involved in this study was 14. Based on these parameters, G*Power calculated the required sample size to be 194. Considering a 10% attrition rate, the estimated minimum sample size was 214 cases. This study actually included 302 cases. This study has been approved by the Ethics Committee of Deyang People’s Hospital (202,204,023 K01).

### 2.2 Data collection

A convenience sampling method was employed to survey 302 older adults across six nursing homes in Deyang City. Prior to the survey, investigators underwent standardized training to familiarize themselves with the study’s specific content, objectives, significance, ethical principles, and precautions. Subsequently, after obtaining permission from nursing home administrators, investigators entered the facilities to conduct the survey. When completing questionnaires, standardized guidance language was used to ensure older adults accurately understood each item and could respond independently. For those unable to self-respond, investigators provided item-by-item explanations, asked clarifying questions, and completed responses on their behalf. Upon completion, investigators immediately verified questionnaire integrity. Participants with incomplete responses were promptly reminded to supplement missing information.

### 2.3 Measures

#### 2.3.1 General information questionnaire

The questionnaire collected information on sex, age, education level, marital status, average monthly family income, drinking alcohol, smoking, diet, exercise, sleep, and number of chronic diseases.

#### 2.3.2 Measurement of frailty

The Tilburg Frailty Indicator (TFI) is used by older adults to self-assess their frailty status. It was developed by Gobbens et al. at Tilburg University in the Netherlands in 2010 (31), with a Cronbach’s alpha coefficient of 0.74. The Chinese version was translated by Xi Xing et al. in 2013 (32), with a Cronbach’s alpha coefficient of 0.686. The scale comprises three dimensions: physical frailty (8 items), psychological frailty (4 items), and social frailty (3 items), totaling 15 items. It uses a dichotomous scoring method (0–1 points), with a total score ranging from 0 to 15 points. A score of ≥5 indicates frailty, with higher scores indicating more severe frailty. In this study, the Cronbach’s alpha coefficient for this scale was 0.666.

#### 2.3.3 Measurement of psychological resilience

The Connor-Davidson Resilience Scale 25 (CD-RISC 25) was used to assess an individual’s level of psychological resilience. The scale was developed by Connor in 2003 (33), and the Cronbach’s alpha coefficient is 0.74. The Chinese version was translated and revised by Yu Xiaonan et al. in 2007 (34), with a Cronbach’s alpha coefficient of 0.91. The scale consists of three dimensions: resilience (13 items), strength (8 items), and optimism (4 items), totaling 25 items. A 5-point Likert scale is used, with scores ranging from 0 to 4, corresponding to ‘never, rarely, sometimes, often, always,’ respectively. The total score ranges from 0 to 100, with higher scores indicating greater psychological resilience. A psychological resilience score below 60 is considered poor, 60–69 is average, 70–79 is good, and 80 or above is excellent. In this study, the Cronbach’s alpha coefficient for this scale was 0.958.

#### 2.3.4 Measurement of QOL

The MOS 36-Item Short Form Health Survey is used to assess the QOL of older adults. This scale was developed by Ware et al. (35) and translated into Chinese by Li et al. (36). It consists of eight dimensions: Physical Functioning (PF), Role-Physical (RP), Bodily Pain (BP), General Health (GH), Vitality (VT), Social Functioning (SF), Role-Emotional (RE), and Mental Health (MH), with a total of 36 items. Each dimension’s score is standardized, with a range of 0 to 100 points, and scores are positively correlated with QOL. In this study, the Cronbach’s alpha coefficient for this scale was 0.918.

### 2.4 Data analysis

Statistical analysis was conducted using SPSS 26.0. The exploratory factor analysis method in the Harman single-factor test was adopted for the common method deviation test. The P–P plot and histogram were combined to determine that the data were approximately normally distributed. Descriptive analysis of the data was performed using the mean ± standard deviation. Pearson correlation analysis was used to test the correlations among frailty, psychological resilience, and QOL. The mediating effect of psychological resilience between frailty and QOL was analyzed using Model 4 (Test Simple Mediating) in Hayes’ PROCESS 4.1 macro program (37). In this study, the total QOL scores for older adults in nursing homes showed statistically significant differences in terms of age, average monthly family income, exercise, and number of chronic diseases (see Table 1). Therefore, these four variables were used as control variables. All tests used the bias-corrected percentile Bootstrap method, with 5,000 repeated samples, to calculate the 95% confidence interval. If the confidence interval did not include 0, the mediating effect was considered significant, with *α* = 0.05.

**Table 1:** Comparison of QOL scores among older adults in nursing homes with different characteristics.

Variable	Item	N(%)	QOL (M ± SD)	*t/F*	*p*	*LSD*
Sex	Male	133(44.0)	56.00 ± 12.74	0.730	0.466	
	Female	169(56.0)	54.87 ± 14.01			
Age	60~	174(57.6)	57.11 ± 12.96a	3.976	0.020	a>b, c
	70~	98(32.5)	53.61 ± 14.72b			
	80~	30(9.9)	50.97 ± 10.24c			
Education level	Illiterate	60(19.9)	54.07 ± 13.43	1.908	0.109	
	Primary school	112(37.1)	53.59 ± 13.85			
	Junior high school	93(30.8)	57.52 ± 12.33			
	High school or technical secondary school	18(6.0)	54.40 ± 16.96			
	College degree or above	19(6.3)	60.31 ± 11.37			
Marital status	Married	265(87.7)	55.62 ± 13.37	1.098	0.335	
	Widowed	28(9.3)	52.05 ± 14.16			
	Divorce	9(3.0)	58.20 ± 13.78			
Average monthly family income	<2000	51(16.9)	51.75 ± 12.72a	5.354	0.005	a,b<c
	2000–5,000	86(28.5)	53.22 ± 15.43b			
	>5,000	165(54.6)	57.60 ± 12.17c			
Drinking alcohol	Never	199(65.9)	55.09 ± 13.47	0.190	0.827	
	Sometimes	70(23.2)	55.59 ± 13.72			
	Often	33(10.9)	56.60 ± 13.10			
Smoking	No/Quit smoking	230(76.2)	54.92 ± 13.83	−1.033	0.302	
	Yes	72(23.8)	56.80 ± 12.18			
Diet	A reasonable mix of meat and vegetables	242(80.1)	55.68 ± 14.05	1.376	0.254	
	Mainly meat	39(12.9)	55.98 ± 8.37			
	Mainly vegetarian	21(7.0)	50.69 ± 13.68			
Exercise	Very few	75(24.8)	52.14 ± 13.23a	4.032	0.019	a<c
	Sometimes	107(35.4)	55.04 ± 13.68b			
	Often	120(39.7)	57.67 ± 13.05c			
Sleep	Normal	124(41.1)	56.24 ± 12.83	1.108	0.332	
	Sometimes insomnia	111(36.8)	55.66 ± 14.93			
	Frequent insomnia	67(22.2)	53.26 ± 11.90			
Number of chronic diseases	0	6(2.0)	65.89 ± 11.94a	4.457	0.012	a, b>c
	1 ~ 2	242(80.1)	55.98 ± 13.32b			
	≥3	54(17.9)	51.46 ± 13.35c			

QOL, quality of life.

## 3 Results

### 3.1 General information on older adults in nursing homes

Among the 302 study participants, 133 were men (44.0%) and 169 were women (56.0%); The average age was 69.31 ± 6.78 years (range: 60–89 years); the majority of participants (87.7%) had an educational attainment of junior high school or below; the majority of participants were married (87.7%); over half of the participants (54.6%) had average monthly family income exceeding 5,000 yuan; 65.9% of participants never drank alcohol, and 76.2% had no smoking habits or had quit smoking; 80.1% of participants believed their diet was balanced between meat and vegetables; 39.7% of participants exercised regularly; 41.1% of participants believed their sleep was normal; the majority of participants (80.1%) were older adults and had 1–2 chronic diseases.

### 3.2 Comparison of QOL scores among older adults in nursing homes with different characteristics

This study used two independent sample t-test and one-way analysis of variance to compare the total QOL scores of older adults in nursing homes with different characteristics. The results showed that there were statistically significant differences (*p* < 0.05) in the total QOL scores of older adults in nursing homes with different ages, average monthly family income, exercise, and number of chronic diseases. As shown in Table 1.

### 3.3 Common method biases test

Exploratory factor analysis was used in Harman’s single-factor test to ensure the reliability and accuracy of the data. The results showed that there were 19 factors with characteristic root values greater than 1 without rotation, and the variance explained by the first factor was 22.49%, which was below the critical standard of 40%, indicating that there was no serious common method bias in the data of this study (38).

### 3.4 Scores and correlation analysis of frailty, psychological resilience, and QOL among older adults in nursing homes

Table 2 shows the mean, standard deviation, and correlation of all study variables. Frailty was negatively correlated with psychological resilience (*r* = −0.365, *p* < 0.01) and QOL (*r* = −0.299, *p* < 0.01) in older adults in nursing homes; psychological resilience was positively correlated with QOL (*r* = 0.450, *p* < 0.01).

**Table 2:** Scores and correlation analysis of frailty, psychological resilience, and QOL among older adults in nursing homes.

Variable	M ± SD	Frailty	Psychological resilience	QOL
Frailty	4.12 ± 2.76	1		
Psychological resilience	62.70 ± 17.27	−0.365^**^	1	
QOL	55.37 ± 13.46	−0.299^**^	0.450^**^	1

**p < 0.01. QOL, quality of life.

### 3.5 The mediating effect of psychological resilience between frailty and QOL among older adults in nursing homes

To test Hypothesis 2, the Model 4 test in the PROCESS 4.1 macro program was used to examine the mediating effect of psychological resilience between frailty and QOL. After controlling for the effects of age, average monthly family income, exercise, and number of chronic diseases, frailty in older adults in nursing homes negatively predicted QOL (β = −0.224, *p* < 0.001) and psychological resilience (β = −0.247, *p* < 0.001); psychological resilience positively predicted QOL among older adults in nursing homes (β = 0.379, *p* < 0.001). The direct effect of frailty on QOL was significant (β = −0.131, *p* < 0.05). As shown in Table 3.

**Table 3:** The mediating effect of psychological resilience between frailty and QOL among older adults in nursing homes.

Variable	Model 1		Model 2		Model 3	
	QOL		Psychological resilience		QOL	
	β	*t*	β	*t*	β	*t*
Age	−0.161	−1.928	0.043	0.558	−0.177	−2.267^*^
Average monthly family income	0.171	2.273^*^	0.248	3.531^***^	0.077	1.070
Exercise	0.072	0.975	0.237	3.429^**^	−0.018	−0.250
Number of chronic diseases	−0.207	−1.485	−0.436	−3.363^**^	−0.041	−0.309
Frailty	−0.224	−3.742^***^	−0.247	−4.412^***^	−0.131	−2.255^*^
Psychological resilience	——	——	——	——	0.379	6.499^***^
*R^2^*	0.362		0.493		0.490	
*Adj R^2^*	0.131		0.243		0.240	
*F*	8.920^***^		19.024^***^		15.510^***^	

^*^p < 0.05, ^**^p < 0.01, ^***^p < 0.001. QOL, quality of life.

**Figure 2:**
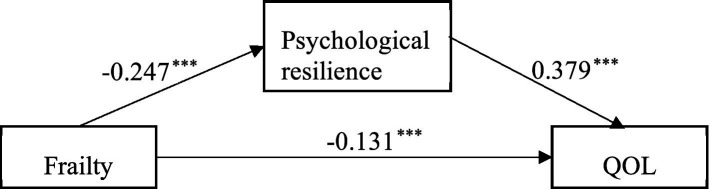
The mediating effect of psychological resilience between frailty and QOL. ^***^ Indicating the coefficient of the path is significant (*p* < 0.001). QOL, quality of life.

Further analysis using the bias-corrected Bootstrap method, with 5,000 repeated samples, revealed that frailty has a significant indirect effect on the QOL of older adults in nursing homes through psychological resilience: β = 0.093, SE = 0.025, 95% CI = [−0.150, −0.050]. The direct effect of frailty (−0.131) and the mediating effect of psychological resilience (−0.093) account for 58.5% and 41.55% of the total effect, respectively. Therefore, the mediating effect of psychological resilience on frailty and QOL is supported in Hypothesis 2. As shown in Table 4 and Figure 2.

**Table 4:** Bootstrap test of the mediating effect of psychological resilience on frailty and QOL among older adults in nursing homes.

Variable	Path	Standardized coefficient	Standard error	*t*	*p*	95% CI
Total effect	Frailty → QOL	−0.224	0.060	−3.742	<0.001	−0.342, −0.106
Direct effect	Frailty → QOL	−0.131	0.058	−2.225	0.025	−0.245, −0.017
Indirect effect	Frailty → Psychological resilience → QOL	−0.093	0.025	——	——	−0.150, −0.050

Bootstrap samples 5,000. QOL, quality of life.

## 4 Discussion

Frailty is a significant burden in the aging process, not only affecting the QOL of older adults but also imposing additional financial burdens on families. This study conducted a cross-sectional survey of older adults in nursing homes in Sichuan, China, to investigate the current status of their QOL and the influence of general demographic factors on QOL. It also explored the relationship between frailty, psychological resilience, and QOL, aiming to provide targeted guidance for improving the QOL of older adults in nursing homes and promoting healthy aging.

In this study, older adults in nursing homes had a QOL score of (55.37 ± 13.46), which was similar to the results of similar studies (55.33 ± 8.37) (39), all were lower than the QOL scores of community-dwelling older adults (40). A 27-year longitudinal study shows that the QOL of older adults declines sharply after they move into nursing homes (41). The reasons for this are twofold. On the one hand, nursing homes are typically managed collectively, limiting personal space, while community-dwelling older adults generally have independent living environments and can arrange their own lives. On the other hand, older adults in nursing homes have fixed social circles and receive few visitors, while community-dwelling older adults are able to maintain their existing social relationships. At the same time, in Chinese culture, institutionalized care is seen as a lack of family support, and older adults may feel abandoned. In addition, meta-analysis results show that family support can significantly improve physical health, mental state, and social relationships compared to nursing home support (42). In summary, these factors may contribute to lower QOL scores among older adults in nursing homes.

General demographic data analysis shows that older adults aged 60–69 have higher QOL scores than those aged 70 and above, indicating that age is a key factor influencing the QOL of older adults, consistent with previous research findings (43). According to literature reports, as older adults age, their organ functions decline significantly, leading to a gradual decrease in their physiological functions and social adaptability, which in turn affects their QOL (44, 45). The QOL scores of older adults with an average monthly family income of more than 5,000 yuan are higher than those of older adults with an average monthly family income of less than 5,000 yuan, indicating that economic status is a protective factor for the QOL of older adults, which is consistent with the results of previous studies (46). The analysis suggests that older adults with better economic conditions have access to more external resources and support, such as high-quality nutritional supplements and rehabilitation aids, and can participate in more paid entertainment activities to enrich their spiritual lives. High-income older adults have sufficient funds to pay for nursing home fees, thereby enjoying higher levels of nursing services and daily care.

This study also found that older adults in nursing homes who exercised regularly had a higher QOL than those who exercised infrequently. This may be because regular exercise can significantly improve muscle strength, balance, and cardiorespiratory function, thereby slowing down the physiological decline caused by aging (47). A systematic review also pointed out that regular physical activity can significantly improve health-related QOL and well-being for people aged 65 and above (48). In addition, the number of chronic diseases is closely related to the QOL of older adults in nursing homes. This study shows that participants with fewer than two chronic diseases have a higher QOL than those with three or more chronic diseases, which is consistent with the results of similar studies (49). The underlying cause is that chronic diseases lead to the decline of various physiological functions in older adults. Common chronic diseases such as diabetes, hypertension, chronic obstructive pulmonary disease, and arthritis often result in multiple complications and functional impairments, causing damage to multiple systems, including the skin, vision, cardiovascular system, and kidneys. This directly weakens older adults’ ability to perform daily activities and reduces their QOL (50, 51). Moreover, chronic diseases in older adults are typically characterized by complex pathogenesis, difficulty in cure, and long duration, requiring long-term or even lifelong medication. Some participants also need to be hospitalized multiple times, which places a heavy psychological and financial burden on participants and their families. This can lead to older adults adopting a passive or evasive approach to their health issues, further undermining their QOL (52).

Beyond the aforementioned factors, this study failed to identify certain variables traditionally considered associated with QOL. For instance, Lou et al. demonstrated that older adults with lower education levels reported poorer QOL compared to those with higher education (49). However, this study did not reveal a correlation between education level and QOL. This discrepancy may stem from differences in research methodologies, sample characteristics, and other potential confounding factors, warranting further exploration in future investigations. Furthermore, Zhang et al. reported that there is a positive correlation between sleep quality and QOL among older adults (53). In this study, while better sleep quality was associated with an upward trend in QOL among older adults in nursing homes, this trend did not reach statistical significance. This may be attributed to the prevalence of chronic diseases or functional impairments among nursing home residents, where underlying health issues constrain overall improvements in QOL (54). Consequently, even with relatively good sleep quality, QOL may remain at a lower level.

The results indicate that frailty is significantly negatively correlated with QOL, consistent with the findings of Gobbens et al. (55), the more severe the frailty, the poorer the QOL. The reasons for this are twofold. On the one hand, frailty leads to muscle weakness and slower walking speed in older adults in nursing homes, limiting their physical activity and severely affecting their ability to perform activities of daily living (ADL), resulting in an inability to meet their self-care needs and thereby reducing their QOL. Related studies indicate that (56, 57), frail older adults often find themselves in a state of abnormal aging prior to illness, making it difficult for them to effectively utilize environmental resources to maintain their health. They often need to rely on others for care, and their impaired self-care abilities further reduce their QOL. On the other hand, frailty, as a complex geriatric syndrome, is often accompanied by multiple chronic diseases, which not only exacerbate the physical suffering of older adults, but also increase their medical and economic burdens and induce negative emotions such as anxiety and depression, significantly reducing their QOL (58, 59). Serrano et al. (60) also pointed out that frail older adults in nursing homes suffer from significantly impaired QOL due to their high vulnerability, multiple coexisting diseases, and high incidence of disability. Therefore, frailty causes older adults to face long-term suffering from disease and poor prognosis, and makes them prone to negative emotions, which affects their overall QOL.

The findings of this study indicate that frailty is significantly negatively correlated with psychological resilience, consistent with the results of Ye et al. (24), suggesting that the more severe the frailty, the poorer the psychological resilience. Studies have shown that increased stressors, decreased functional capacity, isolation, social problems (such as loneliness), and chronic health problems all have a negative impact on the mental health of older adults (61). The greater the degree of frailty in older adults, the more likely they are to experience muscle weakness, slow walking speed, and activity limitations (62), making it difficult for them to participate freely in various activities like healthy individuals. Prolonged physical discomfort and functional limitations can lead to loneliness and social isolation (63), fostering feelings of helplessness and loss (64), thereby weakening psychological resilience. Furthermore, frailty is closely associated with negative emotions such as depression and anxiety (65). Prolonged negative emotions consume substantial psychological resources, making it difficult for older adults to cope with life’s stresses and setbacks (66), thereby reducing their psychological resilience.

Meanwhile, the findings of this study indicate that psychological resilience exhibits a significant positive correlation with QOL. A cross-sectional survey by Gerino et al. among Italian older adults similarly demonstrated that higher levels of psychological resilience correlate with improved QOL (67). Research indicates that psychological resilience, as a key indicator of individual mental health, enables individuals to flexibly cope with adversity, trauma, and threats (33). It mitigates the negative impacts of anxiety and depression, thereby safeguarding mental health and enhancing QOL (68). Research by Xu et al. also indicates that psychological resilience not only directly improves QOL but also indirectly enhances it by improving sleep quality, reducing depression, and mitigating uncertainty in illness (21, 69). Furthermore, Yang et al. found that psychological resilience correlates with older adults’ activities of daily living, physical activity, and overall physical fitness, and together with these factors, contributes to improved QOL (17).

This study found that psychological resilience has a partial mediating effect between the degree of frailty and QOL in older adults in nursing homes, accounting for 41.55% of the total effect. In terms of actual impact, the magnitude of this effect indicates that psychological resilience plays a substantial role. Even if the degree of frailty remains unchanged, improving psychological resilience among older adults has the potential to significantly enhance their QOL. This provides important directional guidance for conducting intervention studies among older adults in nursing homes. This suggests that the degree of frailty in older adults in nursing homes not only directly affects QOL but can also indirectly influence QOL through psychological resilience. These results also validate the buffering mechanism of psychological resilience in the stress process theory (25) and the psychological resilience model of illness (26). When faced with adversity, older adults need to constantly adapt to physiological, psychological, and social decline, which can lead to a decline in self-confidence and sense of control. This can prevent them from effectively mobilizing sufficient psychosocial resources to maintain psychological health and balance, resulting in a decrease in psychological resilience. It is worth noting that low levels of psychological resilience weaken an individual’s ability to mobilize internal and external protective resources, leading to negative coping strategies and exacerbating the impact of health losses on QOL. Conversely, older adults with high psychological resilience have good flexibility and plasticity in terms of psychological dynamics, which can stimulate positive psychological qualities such as resilience, optimism, and self-reliance in participants (70), helping individuals buffer the effects of adverse stimuli and improve their QOL. Therefore, psychological resilience plays an important psychological buffering role between the degree of frailty and QOL in older adults in nursing homes.

## 5 Limitations

The limitations of this study are mainly reflected in the following three aspects. First, due to limitations in manpower, material resources, and financial resources, the study design was a cross-sectional survey. The cross-sectional design prevents establishing causal or directional relationships and only allows for inference of associations. However, the impact of frailty on the QOL of older adults in nursing homes may be a dynamic and long-term process. Future studies may adopt a longitudinal research design to explore its dynamic changes in depth. Second, the pathways through which frailty affects QOL may involve the relevant psychosocial variables, such as social support, self-efficacy, depression, or cognitive functioning, which could provide a more comprehensive understanding of the mechanisms linking frailty and QOL. Third, the exclusive use of self-report measures may have introduced social desirability or recall bias. Although the sample size is adequate, it is limited to a single province in China, which restricts the generalizability of the findings to other cultural or institutional contexts. Future research needs to expand the sample size and broaden the survey area to cover different regions and types of nursing homes in order to enhance the representativeness and reliability of the research results.

## 6 Implications for practice

Despite the aforementioned limitations, this study still provides valuable insights for developing intervention strategies to improve the QOL for older adults in nursing homes. First, at the government level, relevant policies and regulations should be established and improved to safeguard the physical, mental, social, and spiritual health rights of older adults, promote age-friendly smart products and services, and enhance the QOL for older adults. Secondly, frailty is reversible, suggesting that caregivers in older adult care facilities should place importance on screening for frailty indicators in older adults. They should strengthen psychological care and physical exercise for older adults with higher levels of frailty to delay the progression of frailty and thereby improve QOL. Finally, psychological resilience serves as a mediating variable between frailty and QOL, suggesting that enhancing psychological resilience among older adults in nursing homes can improve their QOL. Therefore, it is recommended that older adult care institutions utilize intelligent mental health platforms to provide online psychological counseling services, employing cognitive behavioral therapy, emotional management courses, and other methods to help older adults strengthen their psychological resilience. Additionally, volunteer companionship programs and family support initiatives should be organized to foster a caring social environment for older adults, thereby enhancing the QOL for older adults in nursing homes.

## 7 Conclusion

The results of this study indicate that psychological resilience serves as a mediating variable between frailty and QOL, suggesting that the degree of frailty among older adults in nursing homes not only directly impacts QOL but also indirectly influences it through psychological resilience. The findings validate the buffering mechanism of psychological resilience as proposed in the stress process theory and the disease psychological resilience model, providing a basis for developing strategies to improve the QOL of older adults in nursing homes.

## Data Availability

The raw data supporting the conclusions of this article will be made available by the authors, without undue reservation.
